# Estimating infection fatality risk and ascertainment bias of COVID-19 in Osaka, Japan from February 2020 to January 2022

**DOI:** 10.1038/s41598-023-32639-9

**Published:** 2023-04-04

**Authors:** Tong Zhang, Hiroshi Nishiura

**Affiliations:** School of Public Health, Kyoto University, Yoshida-Konoe-cho, Sakyo-ku, Kyoto, 606-8501 Japan

**Keywords:** Computational biology and bioinformatics, Health care, Disease prevention, Public health, Epidemiology

## Abstract

The present study aimed to estimate the infection fatality risk (IFR) and ascertainment bias of SARS-CoV-2 for six epidemic waves in Japan from February 2020 to January 2022. We used two types of datasets: (i) surveillance-based datasets containing the cumulative numbers of confirmed cases and deaths in each epidemic wave and (ii) seroepidemiological datasets conducted in a serial cross-sectional manner. Smoothing spline function was employed to reconstruct the age-specific cumulative incidence of infection. We found that IFR was highest during the first wave, and the second highest during the fourth wave, caused by the Alpha variant. Once vaccination became widespread, IFR decreased considerably among adults aged 40 years plus during the fifth wave caused by the Delta variant, although the epidemic size of fifth wave was the largest before the Omicron variant emerged. We also found that ascertainment bias was relatively high during the first and second waves and, notably, RT-PCR testing capacity during these early periods was limited. Improvements in the ascertainment were seen during the third and fourth waves. Once the Omicron variant began spreading, IFR diminished while ascertainment bias was considerably elevated.

## Introduction

Coronavirus disease 2019 (COVID-19), caused by severe acute respiratory syndrome coronavirus 2 (SARS-CoV-2), first appeared in Wuhan, China, in late 2019, leading to a pandemic that lasted for more than 2 years. According to the World Health Organization, more than 664 million confirmed cases and 6.7 million deaths have been reported worldwide as of January 22, 2023^1^. Accumulated mutations have led the virus to evolve variants, including Alpha (B.1.1.7), Delta (B.1.617), and Omicron (B.1.1.529)^2, 3^.

The epidemiology of COVID-19 in Japan involved a total of seven distinct waves by July 2022, with each wave eventually suppressed by public health and social measures and vaccination, while new waves were repeatedly induced by the recommencement of social activities and the appearance of novel variants. Of the seven waves, six were fully observed and were divided up in relation to the calendar time: (i) the first wave from February 1, 2020, to June 15, 2020; (ii) the second wave from June 16, 2020, to October 15, 2020; (iii) the third wave from October 16, 2020, to February 28, 2021; (iv) the fourth wave from March 1, 2021, to June 15, 2021; (v) the fifth wave from June 16, 2021, to December 15, 2021; and (vi) the sixth wave from December 16, 2021, to June 30, 2022^4^. The Alpha, Delta, and Omicron variants were the dominant viruses during the fourth, fifth, and sixth waves, respectively^5^.

A certain fraction of infected individuals remains asymptomatic throughout the course of their illness. Ma et al.^6^ conducted a systematic review, indicating that at least 40% of people infected with COVID-19 are asymptomatic, and the proportion of people asymptomatic during an infection with the Omicron variant was estimated to be as high as 80–90%. Thus, a considerable number of infected individuals may not have been diagnosed and reported. A published study estimated that 3.39 billion people (95% CI 3.08–3.63 billion), or 43.9% of the global population, were infected at least once before the spread of Omicron from November 2021^7^. When analysing the risk of death and characterizing virulence, we should not just refer to the case fatality risk (CFR), obtained using the confirmed case count, as the denominator but also pay attention to the infection fatality risk (IFR); that is, the probability of death in the total number of infected individuals^8–12^. Streeck et al. conducted a 7-day seroepidemiological observational study in a small town in Germany after a festival and calculated the IFR^13^. O’Driscoll et al.^14^ and Sorensen et al.^15^ estimated the actual numbers of infected individuals worldwide based on seroepidemiological datasets, and then calculated the age-specific IFRs for different regions. Another research group focused on analysing the relationships between IFR and factors such as age distribution, mean body-mass index, and smoking rate using linear a regression model^16^. Brazeau et al.^17^ estimated the IFR while considering other serological factors, such as delays in seroconversion and seroreversion (i.e., waning of the antibody titer). The estimation of IFR provides key information, but we are also granted an opportunity to estimate the ascertainment bias; that is, the extent of case underestimation caused by a limited ascertainment of infected individuals. During the first wave in China, Nishiura et al.^18^ analysed cases among airline travellers from China to estimate the actual number of infected individuals in the country. However, ascertainment bias has not been consistently monitored thereafter.

In Japan, we have been granted an opportunity to estimate the pandemic ascertainment bias, owing to the existence of reports on a series of cross-sectional seroepidemiological surveys, some of which were conducted independently. Serial cross-sectional data enabled us to estimate the IFR and ascertainment bias over the course of the pandemic. Furthermore, from February 17, 2021, Japan started prioritising a vaccination program using a messenger RNA (mRNA) vaccine. The vaccine stimulates anti-spike antibodies but is believed not to trigger the production of anti-nucleocapsid antibodies^19–21^. Using seroprevalence data for anti-nucleocapsid antibodies, it is possible to grasp the overall magnitude of the respective epidemic waves in Japan.

The purpose of the present study was to estimate the age-dependent IFR and ascertainment bias for each epidemic wave in Japan, using the cross-sectional seroepidemiological data and surveillance-based datasets containing cumulative numbers of confirmed cases and deaths. We aimed to understand variations in the age-specific fatality risk and ascertainment patterns to elucidate the magnitude of different epidemic waves over the course of the pandemic from 2020 to 2022.

## Results

Table 1 shows the estimated incidence of infections for each wave. A trend of increasing incidence in each epidemic wave was commonly seen for all three age groups. Of the five epidemic waves before Omicron, the fifth wave, which was caused by the Delta variant, involved the largest number of cases in each age group. The fitted spline function is shown in Supplementary Fig. S1. The incidence of each epidemic wave was taken to be the difference in the cumulative risks of infection, and the estimates are shown in Supplementary Table S2. Before Omicron, a total of 349,053 people were estimated to have experienced infection in Osaka, and the cumulative risk of infection among adults by the end of fifth wave was 4.7%.

**Table 1:** Cumulative risk of infection and estimated incidence of COVID-19 in Osaka, Japan, 2020–2022.

Wave (calendar time)	Dominated variant^†^	Vaccination^‡^	20–39 years				40–59 years				60 years and older			
			Infection rate (%)	95% CI	Infection number	95% CI	Infection rate (%)	95% CI	Infection number	95% CI	Infection rate (%)	95%CI	Infection number	95% CI
1st (1 Feb 2020–15 Jun 2020)	Wild type	–	0.14	[0.03, 0.58]	2780	[676, 11523]	0.04	[0.01, 0.05]	997	[124, 1158]	0.02	[0.02, 0.42]	513	[495, 12232]
2nd (16 Jun 2020–15 Oct 2020)	Wild type	–	0.89	[0.37, 1.20]	17,605	[7310, 23678]	0.80	[0.62, 1.06]	20,175	[15664, 26756]	0.86	[0.27, 0.90]	25,042	[7707, 26224]
3rd (16 Oct 2020–28 Feb 2021)	Wild type	–	1.17	[0.30, 1.44]	23,008	[5831, 28437]	1.02	[0.79, 1.28]	25,753	[20023, 32204]	0.97	[0.69, 1.04]	28,265	[19999, 30160]
4th (1 Mar 2021–15 Jun 2021)	Alpha (B.1.1.7)	Partly	1.09	[0.33, 1.29]	21,415	[6418, 25501]	0.87	[0.57, 1.10]	21,912	[14386, 27674]	0.76	[0.66, 1.05]	22,070	[19045, 30228]
5th (16 Jun 2021–15 Dec 2021)	Delta (B.1.617.2)	Mostly	2.25	[2.15, 3.00]	44,397	[42527, 59155]	2.27	[1.70, 2.73]	57,168	[42796, 68713]	1.310	[1.13, 1.78]	37,952	[32809, 51554]
6th (16 Dec 2021–31 Jan 2022)	Omicron (B.1.1.529)	+	0.64	[0.58, 1.46]	12,602	[11515, 28864]	0.81	[0.44, 1.20]	20,264	[11141, 30204]	0.33	[0.19, 0.45]	9501	[5648, 13114]

*CI* confidence interval (based on bootstrap method).

^†^The variant of concern that occupied the largest quota in the genome survey during the corresponding wave.

^‡^Qualitative description of the progress of vaccination program.

Table 2 shows the CFR for each wave based on empirically observed case and death data. The CFR among adults aged 20–39 years was the smallest and close to zero, and it was the highest, estimated at 0.04%, during the fourth wave. The CFRs among adults aged 40–59 years were high during the first and fourth waves, calculated at 0.87% and 0.51%, respectively. The CFR among the elderly was always higher than that of the other two age groups and was as large as 16.4% during the first wave and 11.0% in the fourth wave.

**Table 2:** Observed cases and deaths with the estimated case fatality risk (CFR) of COVID-19 in Osaka, Japan, 2020–2022.

Wave	Dominated variant^†^	Vaccination^‡^	Reported confirmed cases			Reported deaths			CFR (%)					
			20–39 years	40–59 years	60 years and older	20–39 years	40–59 years	60 years and older	20–39 years	95% CI	40–59 years	95% CI	60 years and older	95% CI
1st (1 Feb 2020–15 Jun 2020)	Wild type	–	663	572	495	0	5	81	0	[0, 0.58]	0.87	[0.37, 2.03]	16.36	[13.37, 19.88]
2nd (16 Jun 2020–15 Oct 2020)	Wild type	–	4560	2318	1861	0	5	135	0	[0, 0.08]	0.22	[0.09, 0.50]	7.25	[6.16, 8.52]
3rd (16 Oct 2020–28 Feb 2021)	Wild type	–	11,595	9734	10,735	1	17	881	0.01	[0, 0.05]	0.18	[0.11, 0.28]	8.21	[7.70, 8.74]
4th (1 Mar 2021–15 Jun 2021)	Alpha (B.1.1.7)	Partly	19,603	15,684	12,851	7	80	1417	0.04	[0.02, 0.07]	0.51	[0.41, 0.63]	11.03	[10.50, 11.58]
5th (16 Jun 2021–15 Dec 2021)	Delta (B.1.617.2)	Mostly	44,265	26,636	7922	4	70	360	0.01	[0, 0.02]	0.26	[0.21, 0.33]	4.54	[4.11, 5.03]
6th (16 Dec 2021–31 Jan 2022)	Omicron (B.1.1.529)	+	49,395	29,265	13,776	0	4	120	0	[0, 0.01]	0.01	[0.01, 0.04]	0.870	[0.73, 1.04]

*CI* confidence interval (using score confidence interval).

^†^The variant of concern that occupied the largest quota in the genome survey during the corresponding wave.

^‡^Qualitative description of the progress of vaccination program.

On analysing these datasets, the IFR was estimated for each wave (Table 3), and it varied considerably by epidemic wave and age. The IFR during the first wave was the highest among adults aged 40 years and older. Regardless of the epidemic wave, the IFR among the elderly was always higher than that of the other two age groups, consistent with published results^22^. The first wave yielded the highest estimate, 15.8% (95% CI 0.7, 16.4), for IFR among the elderly. When the Delta variant became widespread in the summer of 2021, the elderly was prioritised for vaccination and were fully vaccinated before the end of July 2021, and the IFR was shown to be as low as 0.9% (95% CI 0.7, 1.1). During the sixth wave, caused by the Omicron variant, fatalities among adults aged 20–59 years became extremely rare, and the IFR among the elderly was estimated at 1 3% (95% CI 0.9, 2.5).

**Table 3:** Estimated infection fatality risk (IFR) of COVID-19 in Osaka, Japan, 2020–2022.

Wave	Dominated variant^†^	Vaccination^‡^	IFR (%)					
			20–39 years	95% CI	40–59 years	95% CI	60 years and older	95% CI
1st (1 Feb 2020–15 Jun 2020)	Wild type	–	0	[0, 0]	0.50	[0.43, 4.03]	15.79	[0.66, 16.36]
2nd (16 Jun 2020–15 Oct 2020)	Wild type	–	0	[0, 0]	0.03	[0.02, 0.03]	0.54	[0.52, 1.75]
3rd (16 Oct 2020–28 Feb 2021)	Wild type	–	0	[0, 0.02]	0.07	[0.05, 0.09]	3.12	[2.92, 4.41]
4th (1 Mar 2021–15 Jun 2021)	Alpha (B.1.1.7)	Partly	0.03	[0.03, 0.10]	0.37	[0.29, 0.56]	6.42	[4.69, 7.44]
5th (16 Jun 2021–15 Dec 2021)	Delta (B.1.617.2)	Mostly	0.01	[0.01, 0.01]	0.12	[0.10, 0.16]	0.95	[0.70, 1.10]
6th (16 Dec 2021–31 Jan 2022)	Omicron (B.1.1.529)	+	0	[0, 0]	0.02	[0.01, 0.04]	1.260	[0.90, 2.54]

*CI* confidence interval (using bootstrap method).

^†^The variant of concern that occupied the largest quota in the genome survey during the corresponding wave.

^‡^Qualitative description of the progress of vaccination program.

**Figure 1:**
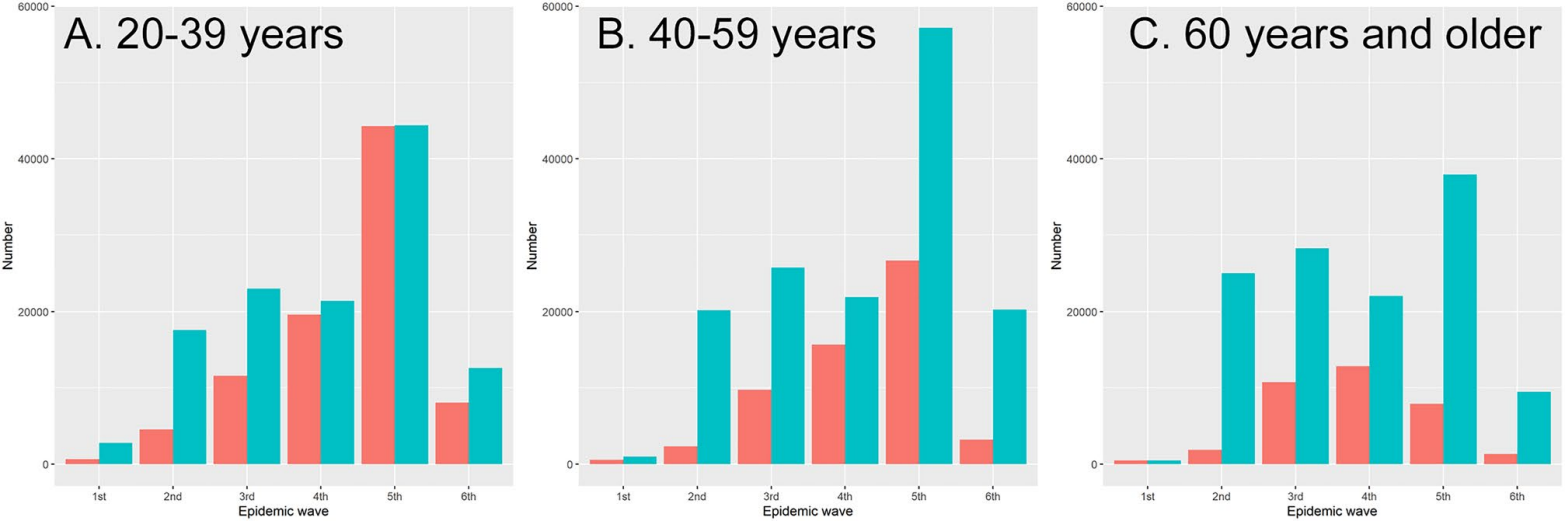
Comparison between observed confirmed cases and estimated number of infections with COVID-19 in Osaka, Japan, 2020–2022. The horizontal axis shows the epidemic wave in Japan from 2020 to 2022, while the vertical axis represents the absolute number of cases. The red bars represent the observed number of confirmed cases, while the blue bars show the estimated number of infections. The left panel (**A**) shows estimates for young adults aged 20–39 years, the middle panel (**B**) for those aged 40–59 years, and the right panel (**C**) shows the elderly aged 60 years and older.

**Figure 2:**
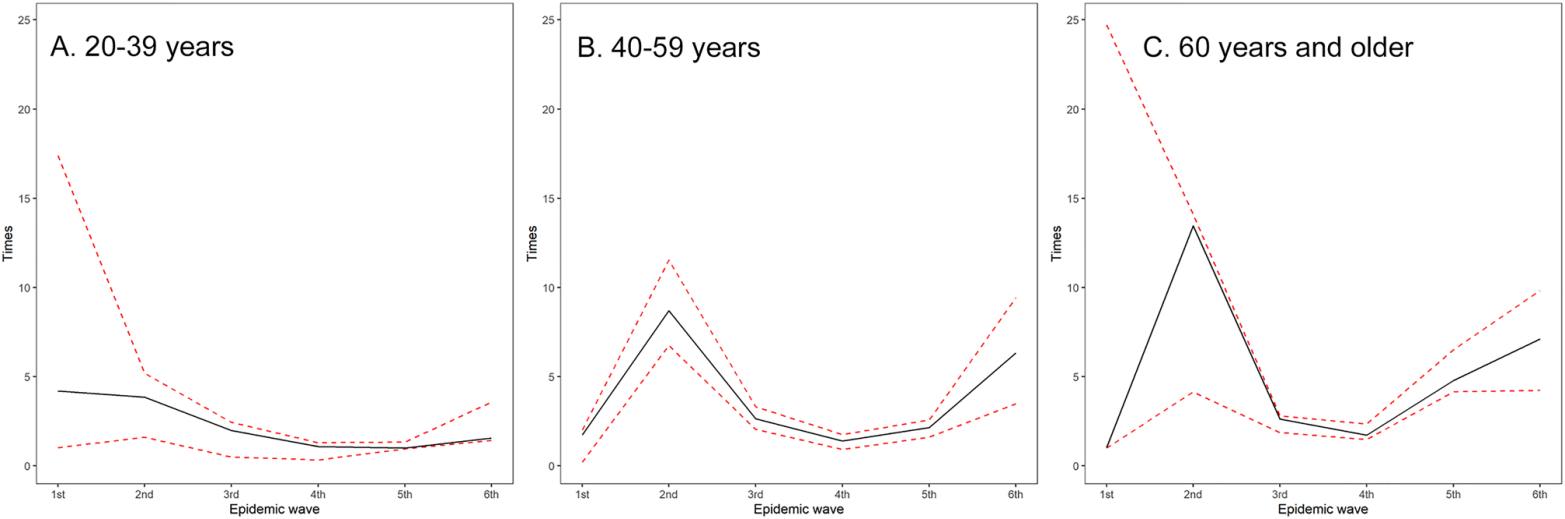
Ascertainment bias estimates by age group and epidemic wave in Osaka, Japan, 2020–2022. The horizontal axis shows the epidemic wave in Japan from 2020 to 2022, and the vertical axis represents the estimated ascertainment bias. Ascertainment bias is expressed as the ratio of the estimated number of infections to the observed number of confirmed cases. The left panel (**A**) shows estimates for young adults aged 20–39 years, the middle panel (**B**) for those aged 40–59 years, and the right panel (**C**) shows estimates for those aged 60 years and older. The solid lines show least square estimates, and the red dashed lines show 95% confidence intervals computed using the bootstrap method.

Figure 1 compares the observed count of confirmed cases against the estimated incidence of infection, which allowed us to estimate the ascertainment bias in Fig. 2 for each epidemic wave. Among adults aged 20–39 years, ascertainment bias was the greatest during the first wave, estimated at 4.2-fold, and the second highest was in the second wave. Ascertainment biases among adults aged 40–59 years and 60 years and older peaked during the second wave, estimated at 8.7- and 13.5-fold, respectively. Ascertainment bias had the lowest estimates during the third and fourth waves, but then abruptly increased during the fifth wave among the elderly and during the sixth (Omicron variant) wave among all adults. Sensitivity analysis of the data of truncation did not alter these observations (Supplementary Fig. S2). Ascertainment biases were qualitatively similar across age groups.

## Discussion

The present study assessed the cumulative risk of infection in Japan using serial cross-sectional seroprevalence data. By obtaining the cumulative risk of infection continuously from the spline model and calculating the CFR in Osaka for each epidemic wave and age group, we were able to compute the IFR by epidemic wave and age group. These procedures enabled us to estimate the ascertainment bias of cases by age group over the 2-year course of the pandemic in a single geographic unit, Osaka, from 2020 to 2022. To the best of our knowledge, the present study is the first to have longitudinally quantified the IFR and ascertainment bias of COVID-19 in Japan.

Five notable take-home messages from this study are as follows: (i) the size of the epidemic before the Omicron variant evolved was the largest during the fifth wave, which was caused by the Delta variant; (ii) IFR was highest during first wave and second highest during the fourth wave, caused by the Alpha variant; (iii) once vaccination became widespread, IFR decreased considerably among adults aged 40–59 years and 60 years and older during the fifth wave; (iv) ascertainment bias was high during the first and second waves but decreased during the third and fourth waves; and (iv) once the Omicron variant emerged, the IFR diminished while ascertainment bias inflated.

Explicitly estimating the size of an epidemic is vital to obtaining a thorough grasp of the epidemiological dynamics. For instance, if we relied on reported confirmed cases (Table 2), the number of Delta-variant-infected people would remain underestimated. Among the elderly, only 7922 confirmed cases were reported during the fifth wave, but actually, as many as 38,000 infections should have taken place. From July to August 2021, the Olympic Games were held in Tokyo, Japan, under a declaration of a state of emergency, and healthcare facilities were overwhelmed with high caseload pressure^23^. Vaccination as well as under-ascertainment due to limited hospital capacities during the course of the fifth wave may have influenced the elevated ascertainment bias among the elderly during the corresponding period.

The IFR was the highest during the first wave, which is understandable, because specific treatments for severe cases were still under development. Notably, the absence of treatments along with the governmental policy that advised patients to remain at home for the first four days of their illness could have had an impact on the IFR^24^. Because COVID-19 started to spread during the winter of 2020, the government considered that differentiating COVID-19 from influenza would be difficult, and thus a 4-day rule was in place by early May 2020. The second highest IFR was seen during the fourth wave in Osaka, which is known to have delayed declaring a state of emergency, and again healthcare facilities in Osaka were overwhelmed with enormous caseloads^25^.

Similarly, ascertainment bias was relatively high during the first and second waves, and it should be noted that RT-PCR testing capacity during these early periods was limited. Nevertheless, improvements were seen during the third and fourth waves, while the Omicron variant elevated the level of ascertainment bias. Infection with the Omicron variant induces general clinically mild symptoms^26^, and the presence of mild and asymptomatic cases can explain the observed finding. The sixth wave was also unique, in that the IFR was also considerably low. Although we cannot be certain, vaccination prior to the Omicron epidemic and the limited virulence of this variant compared with the Alpha and Delta variants may explain this observation.

As an important technical remark, we should discuss the smoothing procedure for the cumulative risk of infection. We employed a smoothing spline model to fit the seroprevalence curve because we were unable to determine the actual infection rate over time without the model. To do so, we assumed that the cumulative risk of infection would share similar time-dependent patterns of increase to the cumulative number of confirmed cases. As we minimised the distance D, we were able to determine the spline parameter *λ*, enabling statistical estimations of IFR and the ascertainment bias. The proposed method could potentially act as an alternative to the prevalence survey used in the United Kingdom that requires repeated monitoring of RT-PCR testing results in the general population^27^.

This study had technical limitations. First, we consistently relied on anti-N antibody data. Although the antibody reflects the incidence of natural infection, we cannot fully and precisely exclude a possible increase due to vaccination (e.g., inactivated vaccines). Nevertheless, more than 98% of vaccinations in Japan involve a messenger RNA vaccine^28^. For simplicity, antibody decay was discarded. Second, the included seroprevalence surveys were inconsistent in terms of sampling areas, sampling methods, and serum testing methods. Rather than these points, the present study valued the presence of seroprevalence data over time and for age groups. Third, the very last survey was conducted in the midst of the Omicron variant epidemic, and thus we had to truncate a part of the observed data; taking into account that only the early stage of the Omicron wave might have led to an underestimation of the IFR because healthcare pressure may have been later elevated. Similarly, ascertainment bias might have been underestimated for the sixth wave, considering that caseloads in and after February 2022 were far greater than those in January 2022. Fourth, excess all-cause deaths possibly related to COVID-19 pandemic were observed during the course of time in Japan^29^, but we simply used confirmed number of deaths to estimate the IFR, because we did not have causally attributed death estimate that could be extracted from estimated excess deaths. Fifth, the present study focused on the parameters of time and age, and underlying comorbidities and other conditions, including obesity, were ignored, despite being known to influence the risk of death from COVID-19^30^.

## Conclusions

The present study successfully estimated the IFR and ascertainment bias in Osaka in a longitudinal manner. Both the IFR and ascertainment bias were high during the first and second waves, and once vaccination became widespread, the IFR decreased considerably among the elderly. The emergence of Omicron led to an abrupt increase in the ascertainment bias, and clarifying that feature is the subject of our ongoing study.

## Methods

### Epidemiological data

We used two different types of datasets: (i) epidemiological data containing the cumulative numbers of confirmed cases and deaths for each epidemic wave, and (ii) seroepidemiological data from surveys conducted in a cross-sectional manner. In the collation of epidemiological data, the present study focused on Osaka Prefecture, the third largest prefecture in Japan. To extract the cumulative number, the daily number of confirmed cases from February 1, 2020, to January 31, 2022, and the specific death dates of deceased individuals were extracted^31^. With respect to the collation of seroepidemiological data, a mixture of five cross-sectional survey datasets was combined^32–35^. Three were nationally representative surveys conducted by the Ministry of Health, Labour, and Welfare of Japan^32, 35^ and yielded age-specific seroprevalence data. The other two sources were cross-sectional studies in Hyogo^33^, the neighbouring prefecture of Osaka and Tokyo, the capital of which experienced a comparably sized epidemic^34^. It should be noted that confirmed cases include reinfected individuals, while the overall risk of infection was maintained to be very small in Japan by the end of 2021.

The first national survey took place in Tokyo, Osaka, and Miyagi from June 1–7, 2020^32^. Subjects were randomly invited to voluntarily provide blood samples, and an antibody against a SARS-CoV-2 nucleocapsid antigen was tested by employing a chemiluminescent microparticle immunoassay with a specificity of 99.6–99.9% (SARS-CoV-2 IgG assay; Abbott). The subsequently conducted survey was an original study carried out in Hyogo from August 6 to October 1, 2020, analysing serum samples in five hospitals and a healthcare foundation^33^. In this study, two different testing methods—(i) a electrochemiluminescence immunoassay (ECLIA) using the Elecsys Anti-SARS-CoV-2 assay and the Cobas e801 module (Roche Diagnostics, Rotkreuz, Switzerland) and (ii) a chemiluminescent enzyme immunoassay (CLEIA)—were both used for detecting IgG against the SARS-CoV-2 nucleocapsid protein, and sera that were positive either by ECLIA or CLEIA were recorded as positive. The third survey took place in Tokyo from September 1, 2020, to March 31, 2021^34^. Participants were randomly selected from outpatient visits to 14 hospitals in Tokyo. Anti-SARS-CoV-2 IgG was analysed using an iFlash 3000 chemiluminescence immunoassay analyser (Shenzhen YHLO Biotech, Shenzhen, China) with an iFlash–SARS-CoV-2 IgG kit, which primarily detects anti-N antibodies. The fourth and fifth surveys were nationally representative surveys conducted in Tokyo, Osaka, Miyagi, Aichi, and Fukuoka from December 3 to 27, 2021, and from February 2 to March 6, 2022, respectively^35^. Subjects were randomly sampled and invited to voluntarily donate blood samples. Anti-nucleocapsid antibodies were measured using Roche’s Elecsys Anti-SARS-CoV-2 system. In addition to antibody-positive results, people with a past history of a confirmed diagnosis of COVID-19 were defined as positive.

At present, the vaccines used in Japan (mRNA vaccine and virus vector vaccine) do not have any viral genome sequences other than the sequence encoding the spike(s) antigen. The production of anti-spike antibodies is induced by both natural infection and vaccination. However, anti-nucleocapsid antibodies are only produced after a natural infection. Thus, the number of infected individuals can be measured by examining the presence of anti-nucleocapsid antibodies. Table 4 summarises the above-mentioned surveys and serological testing methods.

**Table 4:** Five seroepidemiological surveys of COVID-19 conducted in Japan, 2020–2022.

Study period	Place (prefecture)	Testing method of anti-nucleocapsid antibody	Sample size (persons)	Positive cases (persons)	Ref.
June 1–7, 2020	Tokyo, Osaka, Miyagi	Chemiluminescent microparticle immunoassay	7950	23	^32^
August 6–October 1, 2020	Hyogo	ECLIA and CLEIA	10,377	44	^33^
September 1, 2020–March 31, 2021	Tokyo	iFlash 3000 chemiluminescence immunoassay	23,234	242	^34^
December 3–27, 2021	Tokyo, Osaka, Miyagi, Aichi, Fukuoka	Elecsys	8147	204	^35^
February 2–March 6, 2022	Tokyo, Osaka, Miyagi, Aichi, Fukuoka	Elecsys	8149	348	^35^

For the population census, we referred to the national population survey of 2020, focusing on Osaka Prefecture^36^.

### Statistical estimation of cumulative incidence

We entered the data for the cumulative incidence over time from June 2020 to February 2022 into the statistical model. To ensure the smoothness of the fitted cumulative incidence, a smoothing spline function was employed to identify patterns in the cumulative risk of infection over the course of time. We used serial cross-sectional data from five serum surveys and assumed that each survey result represented the seroprevalence in the middle of each month; namely, June 15, 2020; August 15, 2020; December 15, 2020; December 15, 2021; and February 15, 2022. Because continuously observed data were missing, a smoothing spline model was fitted to the seroprevalence data to deal with the data in a discrete manner (i.e., every 15 days) for the period from June 15, 2020, to February 15, 2022. The calendar time was used as predictor variable and the knots for the spline were evenly spaced in the conducting times of the five serum surveys. The smoothing parameter *λ* was determined by comparing the spline model solution against the cumulative count of confirmed cases. That is, let ${f}_{i}\left(t\right)$ be the cumulative incidence, as predicted by the smoothing spline, for wave *i* at calendar time *t*, and similarly, let ${c}_{i}\left(t\right)$ be the cumulative number of confirmed cases during wave *i* at reporting date *t*. We computed the following distance *D*:

$$D=\sum_{i}\left\{\sum_{t}{\left[\frac{{f}_{i}\left(t\right)}{{w}_{i}}-{c}_{i}\left(t\right)\right]}^{2}\right\},$$

where *w*_i_ is the ascertainment bias calculated as ${w}_{i}={f}_{i}({t}_{i})/{c}_{i}({t}_{i})$, and where *t*_i_ was the last date of *i*-th epidemic wave. Because the present study handled datasets covering more than 2 years, we discarded the small time-lag between seroconversion and reporting date. In a range from 0 to 1 (e.g., 0.001, 0.002,.…, 1), we calculated *D* and found *λ* that minimised error. Bootstrap-based 95% confidence intervals (CI) were computed by resampling positive individuals from each survey 1000 times.

### Estimation of IFR and ascertainment bias

Given the smoothed incidence of infection, we estimated the IFR and ascertainment bias up to the end of January, 2022, that is, for all six waves. Before estimating the IFR, we first analysed the confirmed case and death datasets by January 31, 2022, to estimate CFR by age group for each epidemic wave *i*,

$${CFR}_{i}=\frac{{M}_{i}}{{C}_{i}},$$

where *M*_i_ and *C*_i_ are cumulative numbers of deaths and confirmed cases, respectively, of wave *i*. Notably, CFR_i_ was modelled as a function of epidemic wave *i*. Furthermore, we discarded small time-lags between the date of reporting and the date of death because the number of cases between different epidemic waves tended to be very small. The 95% CI was computed using the Wilson score interval. Subsequently, we calculated the IFR using death count data and estimated the cumulative incidence of wave *i*, *F*_i_, that is,

$${IFR}_{i}=\frac{{M}_{i}}{{F}_{i}},$$

Finally, the ascertainment bias *B*_i_ was subsequently computed as the ratio of the total to ascertained (confirmed) number of infected individuals, that is,

$${B}_{i}=\frac{{F}_{i}}{{C}_{i}}.$$

Notably, *w*_i_ was estimated from the first to fifth waves by minimising the above-mentioned *D*, that is, *w*_i_ = *B*_i_ for *i* = {1,2,3,4,5}, but *B*_i_ was uniquely calculated using this equation for the sixth wave. As for the sixth wave (Omicron wave), we analysed the cases observed by January 15, 2022, while the cumulative risk of infection using the spline model was modelled until January 31, 2022, allowing 2 weeks for humoral immune responses to be established^37^. This was in the midst of the sixth wave, but the truncation was required to correspond to the timing of the fourth national seroepidemiological survey, which took place during the course of the sixth wave. As part of the sensitivity analysis, we varied the date of truncation from January 10 to January 20.

## Supplementary Information

Supplementary Legends.

Supplementary Figure S1.

Supplementary Figure S2.

Supplementary Table S1.

Supplementary Table S2.

## Data Availability

The systematically collected datasets are listed in Supplementary Table S1.
