# Supplementary material for: Estimating infection fatality risk and ascertainment bias of COVID-19 in Osaka, Japan from February 2020 to January 2022
Source: Sci Rep. 2023 Apr 4;13:5540. doi: 10.1038/s41598-023-32639-9 (PMC10072030; doi:10.1038/s41598-023-32639-9)
Supplement: Supplementary file 1 — Supplementary Legends. [file 41598_2023_32639_MOESM1_ESM.docx]

**Figure S1. Seroprevalence curves of COVID-19 fitted by smoothing spline in Japan, 2020**–**2022**

The horizontal axis shows the calendar time, while the vertical axis represents the fraction of immune individuals, which we assumed reflect the fraction of infected individuals. The left panel (A) shows the estimates for young adults aged 20–39 years, the middle panel (B) for those aged 40–59 years, and the right panel (C) shows the elderly aged 60 years and older. The solid lines show the seroprevalence curve fitted using smoothing spline, and the red dashed lines show 95% confidence intervals computed using the bootstrap method. White circles represent the observed values from published seroprevalence surveys, while blue triangles show the point estimates from which we computed the infection fatality risk and ascertainment bias for six different epidemic waves in Japan.

**Figure S2. Sensitivity analysis for three age groups**

The horizontal axis shows the date at which the observations of cases were truncated, varying from January 10 to January 20, 2022, and the vertical axis represents the estimated ascertainment bias. Ascertainment bias is expressed as the ratio of the estimated number of infections to the observed number of confirmed cases. The left panel (A) shows estimates for young adults aged 20–39 years, the middle panel (B) for those aged 40–59 years, and the right panel (C) shows the elderly aged 60 years and older. The solid lines show the least square estimates, and the red dashed lines show 95% confidence intervals computed using the bootstrap method.
