# Supplementary figures and images for: Estimating infection fatality risk and ascertainment bias of COVID-19 in Osaka, Japan from February 2020 to January 2022
Source: Sci Rep. 2023 Apr 4;13:5540. doi: 10.1038/s41598-023-32639-9 (PMC10072030; doi:10.1038/s41598-023-32639-9)

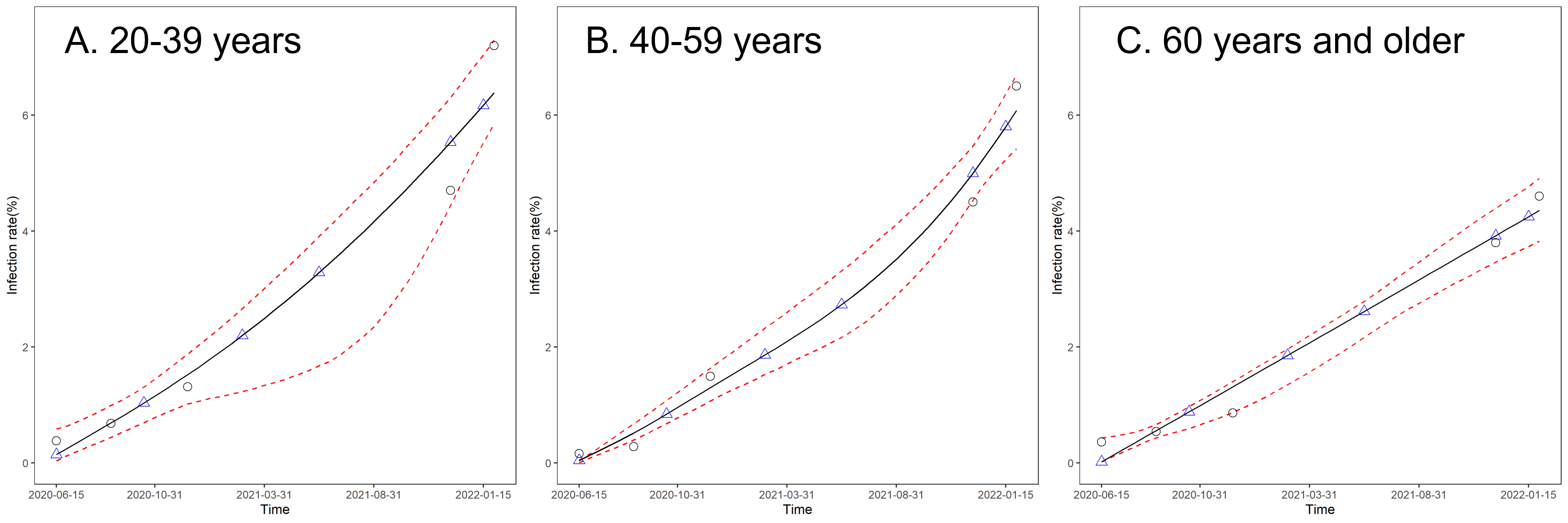

Supplement: Supplementary file 2 — Supplementary Figure S1. [file 41598_2023_32639_MOESM2_ESM.tif]

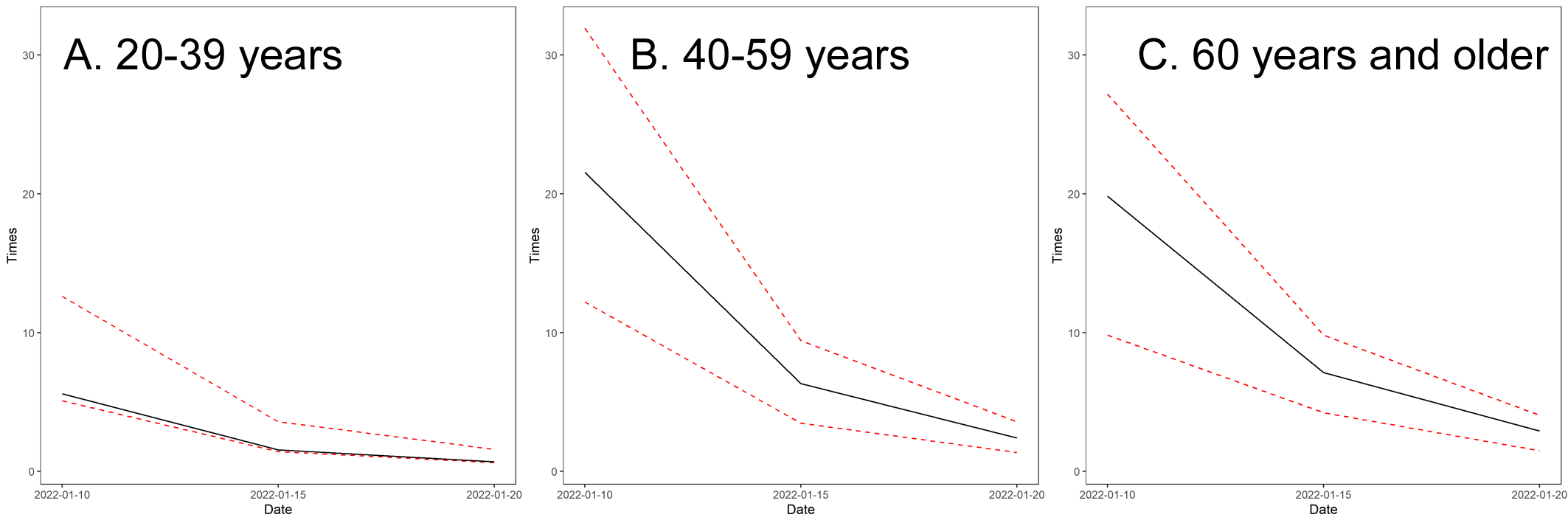

Supplement: Supplementary file 3 — Supplementary Figure S2. [file 41598_2023_32639_MOESM3_ESM.tif]
