# Supplementary material for: Estimating infection fatality risk and ascertainment bias of COVID-19 in Osaka, Japan from February 2020 to January 2022
Source: Sci Rep. 2023 Apr 4;13:5540. doi: 10.1038/s41598-023-32639-9 (PMC10072030; doi:10.1038/s41598-023-32639-9)
Supplement: Supplementary file 5 — Supplementary Table S2. [file 41598_2023_32639_MOESM5_ESM.docx]

Table S2. Cumulative incidence and estimated number of infections of COVID-19 in Osaka, Japan, 2020-22

| Wave | Dominated variant^†^ | Vaccination^‡^ | Cumulative infection rate (%) | | | | | | Cumulative number of infections | | | | | |
| --- | --- | --- | --- | --- | --- | --- | --- | --- | --- | --- | --- | --- | --- | --- |
|  |  |  | 20-39 years | 95% CI^§^ | 40-59 years | 95%CI | 60 years and older | 95%CI | 20-39 years | 95% CI^§^ | 40-59 years | 95%CI | 60 years and older | 95%CI |
| 1^st^ (1 Feb 2020-15 Jun 2020) | Wild type | - | 0.14 | [0.03 , 0.58] | 0.04 | [0.01 , 0.05] | 0.02 | [0.02 , 0.42] | 2780 | [676 , 11523] | 997 | [124 , 1158] | 513 | [495 , 12232] |
| 2^nd^ (16 Jun 2020-15 Oct 2020) | Wild type | - | 1.03 | [0.71 , 1.34] | 0.84 | [0.65 , 1.09] | 0.88 | [0.60 , 0.97] | 20385 | [13969 , 26477] | 21173 | [16424 , 27371] | 25555 | [17313 , 28152] |
| 3^rd^ (16 Oct 2020- 28 Feb 2021) | Wild type | - | 2.20 | [1.20 , 2.72] | 1.86 | [1.51 , 2.33] | 1.85 | [1.36 , 1.97] | 43393 | [23650 , 53620] | 46926 | [37953 , 58681] | 53820 | [39415 , 57325] |
| 4^th^ (1 Mar 2021-15 Jun 2021) | Alpha (B.1.1.7) | Partly | 3.28 | [1.58 , 3.95] | 2.73 | [2.17 , 3.35] | 2.61 | [2.16 , 2.81] | 64808 | [31178 , 77871] | 68838 | [54814 , 84569] | 75889 | [62755 , 81648] |
| 5^th^ (16 Jun 2021- 15 Dec 2021) | Delta (B.1.617.2) | Mostly | 5.53 | [4.39 , 6.37] | 5.00 | [4.54 , 5.55] | 3.92 | [3.44 , 4.43] | 109206 | [86620 , 125667] | 126006 | [114520 , 139985] | 113841 | [100030 , 128536] |
| 6^th^ (16 Dec 2021-31 Jan 2022) | Omicron (B.1.1.529) | + | 6.17 | [5.49 , 7.13] | 5.80 | [5.25 , 6.37] | 4.25 | [3.73 , 4.83] | 121808 | [108303 , 140788] | 146270 | [132253 , 160653] | 123343 | [108462 , 140428] |

^†^The variant of concern that occupied the largest quota of genome survey during the corresponding wave; ^‡^Qualitative description of the progress of vaccination program; ^§^CI, confidence interval (using bootstrap method).
